# Multistakeholder Consultations on Programmatic Progress, Challenges, and Policy Priorities for Lymphatic Filariasis Elimination in India

**DOI:** 10.4269/ajtmh.26-0054

**Published:** 2026-08-27

**Authors:** Muhammed Jabir, Raja Jeyapal Dinesh, Adinarayanan Srividya, Anju Viswan, Manikandan Kishanthini, Richa Sharma, Manju Rahi

**Affiliations:** ^1^ Indian Council of Medical Research-Vector Control Research Centre, Indira Nagar, Puducherry, India;; ^2^ Women’s Collective Forum, New Delhi, India

## Abstract

This report presents the key proceedings and outcomes of three regional consultative workshops convened to review progress, identify challenges, and propose strategic actions for lymphatic filariasis elimination in India. The workshops were organized by the Indian Council of Medical Research-Vector Control Research Centre, Puducherry in collaboration with the Women’s Collective Forum, New Delhi from June 2025 to August 2025. Consultations were held in three endemic states representing different regions of India (Uttar Pradesh, Gujarat, and Tamil Nadu), bringing together stakeholders from the national program, the WHO, donor agencies, nongovernmental organizations, academia, and research institutions. The consultations revealed that the nature of challenges varied considerably by region. Common challenges included gaps in mass drug administration (MDA) coverage and associated reporting and the persistence of never-treated populations. Shortfalls in diagnostic availability, surveillance capacity, and morbidity management services were also reported across regions. Rapid urbanization and an increase in informal settlements with poor facilities were identified as a cause of concern. Recommended programmatic actions included strengthening field supervision, real-time MDA monitoring, targeted strategies for never-treated and high-refusal populations, and improving diagnostic supplies and surveillance capacities. Context-specific interventions included migrant-focused surveillance and treatment, urban microplanning, and intensified post-MDA surveillance in areas that have passed transmission assessment surveys. Long-term priorities included strengthening intersectoral coordination, scaling up molecular xenomonitoring for vector surveillance, and expanding public–private partnerships for morbidity management. Thus, the workshop enabled the identification and discussion of the critical region-specific challenges and also helped to provide operationally feasible strategies to address these issues.

## INTRODUCTION

Lymphatic filariasis (LF) is a disabling neglected tropical disease spread through the bites of infective mosquitoes. It remains a major public health challenge in many parts of the world, especially in Asian, Pacific, and African regions.^1^ Since the launch of the Global Program to Eliminate Lymphatic Filariasis (GPELF) in 2000, endemic countries, including India, have been striving to achieving the global goal of LF elimination by 2030. India initiated its national LF elimination program in 2004, adopting GPELF-recommended strategies: 1) mass drug administration (MDA) in endemic districts and 2) morbidity management and disability prevention (MMDP) for affected individuals.^2^

## BACKGROUND

In 2007, MDA with two drugs (diethylcarbamazine and albendazole [DA]) was implemented in all endemic districts. In 2018, to accelerate elimination, a triple-drug regimen with (ivermectin, diethylcarbamazine, and albendazole [IDA]) was introduced. Subsequently, in 2023, a five-pronged strategy was used that included biannual mission-mode MDAs, administering antifilarial drugs twice a year to cover endemic areas in a phased manner at the block level. This strategy also recommended involvement of medical colleges in MMDP care, integrated vector control, collaboration between ministries, and innovative approaches to enhance the elimination efforts.^2^ These efforts have been reinforced by support from national and international partners, including the WHO.^3^^,^^4^

Despite significant progress, India continues to carry the world’s highest LF burden, with an estimated 404 million people at risk in 348 districts in 20 states and union territories.^5^^,^^6^ As of 2024, 164 (47.1%) districts across 14 states have reported >1% microfilaria (Mf) rate and are implementing MDA. Although 141 (40.5%) districts passed the first transmission assessment survey (TAS) and stopped MDA, 43 (12.4%) other districts are at various stages of pre-TAS/TAS or the IDA Impact Survey. In terms of disease burden, the country recorded approximately 620,345 cases of lymphoedema and 121,486 cases of hydrocele.^7^ MDA coverage gaps in many districts highlight programmatic gaps that must be addressed to achieve elimination.^8^^–^^10^

The Indian Council of Medical Research (ICMR)-Vector Control Research Centre (VCRC), Puducherry is a premier institute under the ICMR, Ministry of Health and Family Welfare mandated to work on LF and a WHO collaborating center for research and training in LF. The institute has made significant contributions toward LF elimination programs in India, such as developing sampling strategies for molecular xenomonitoring for post-MDA surveillance; mapping of endemic areas; assessing safety, efficacy, and acceptability of IDA versus DA; and creating a monitoring and evaluation protocol for accelerated MDA with IDA.^11^^,^^12^ The Women’s Collective Forum (WCF) is a platform dedicated to advancing women’s empowerment, leadership, and collective action, particularly among marginalized and grassroots communities.

## REGIONAL WORKSHOPS

**Figure 1.**
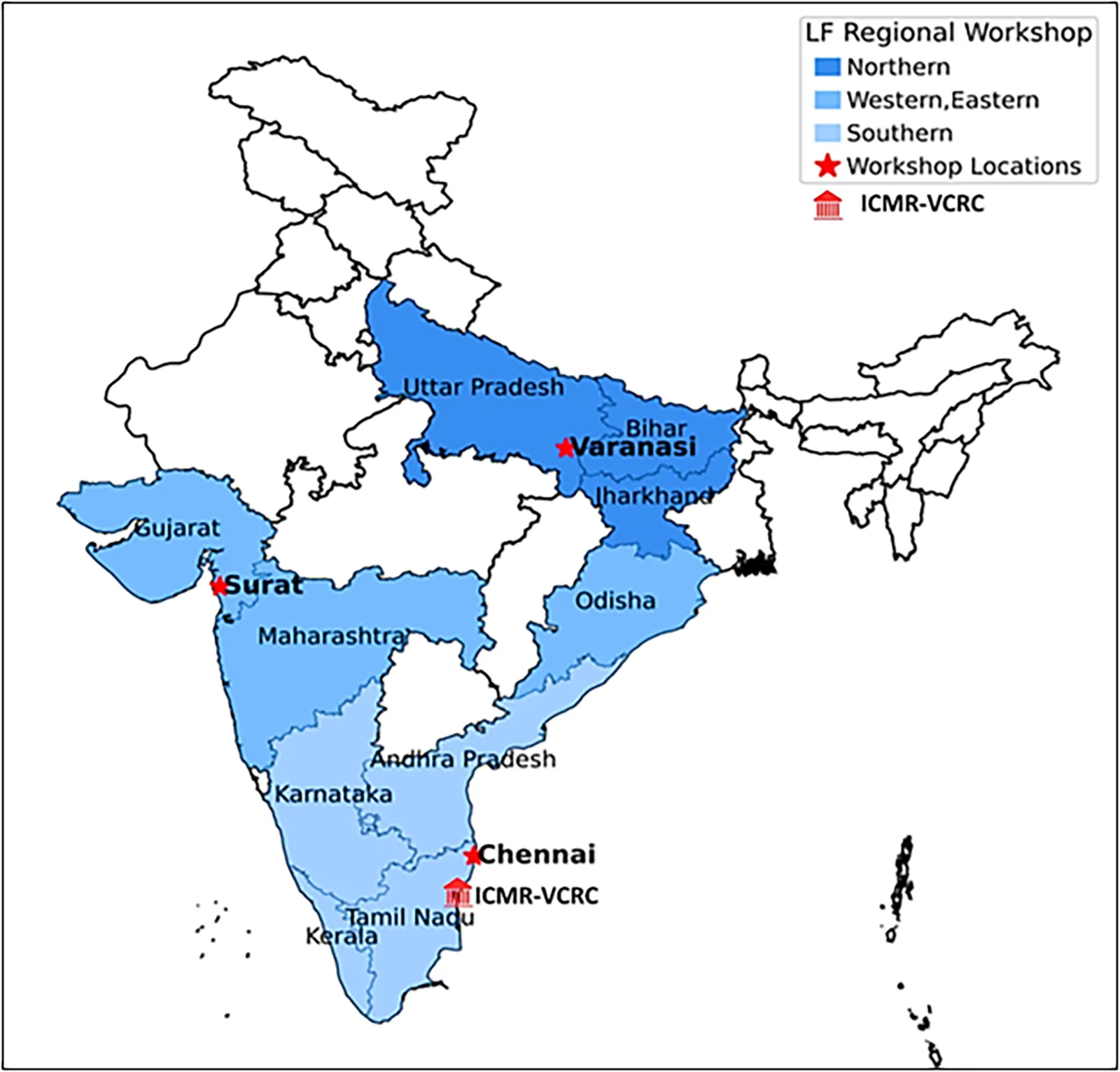
Workshop locations and the states represented in the three zones. ICMR-VCRC = Indian Council of Medical Research-Vector Control Research Centre; LF = lymphatic filariasis.

ICMR-VCRC collaborated with the WCF to conduct three workshops on LF between June and August 2025: in Varanasi in Uttar Pradesh (*n* = 35), in Surat in Gujarat (*n* = 34), and in Chennai in Tamil Nadu (*n* = 32), covering the northern, western/eastern, and southern regions of the country, respectively (Figure 1). Altogether, more than 100 participants from 11 states participated, including officials from the NCVBDC, officials from the Regional Office of Health and Family Welfare, officials from state/district vector-borne disease (VBD) control programs, officials from the WHO country office in India, development partners, municipal authorities, academia, nongovernmental organizations (NGOs), donors, and researchers.

These consultations provided a platform for evidence-based deliberations among stakeholders representing endemic states at different stages of LF elimination. The consultations opened with welcome remarks by Manju Rahi, Director of the ICMR-VCRC, who outlined the objectives of the workshops. Representatives from the NCVBDC presented the current status of the national elimination program. The WHO representatives shared perspectives on aligning India’s efforts with the WHO 2030 road map, highlighting state-wise progress and key programmatic priorities. Partners, including the Gates Foundation, Project Concern International (PCI), and the Lepra Foundation, shared their field experiences. After these deliberations, state-level progress was presented by program representatives followed by expert technical inputs and discussions on key programmatic challenges.

## STATUS OF LF BURDEN AND PROGRESS IN PARTICIPATING STATES

The LF status across participating states reflected considerable regional heterogeneity in disease burden and program progress (Table 1). Although a substantial number of districts have passed TAS and stopped MDA, many continue drug administration. Northern states, including Bihar, Jharkhand, and Uttar Pradesh, carry a disproportionately high burden. All districts in Bihar and Jharkhand are endemic. Bihar has documented more than 150,000 lymphoedema cases and 20,000 hydrocele cases. The state had mapped 199 high-priority blocks based on night blood surveys (NBS), of which 44 blocks were identified with either Mf rates above 5% or MDA coverage below 45%. In Jharkhand, nine districts implemented the triple-drug regimen. The state had recorded more than 50,000 lymphoedema cases and 40,000 hydrocele cases. Uttar Pradesh has 51 endemic districts, with 100,000 lymphoedema cases and 10,000 hydrocele cases, and it achieved 73% coverage during the MDA round in 2024.

**Table 1:** State-specific operational challenges/program shortcomings in lymphatic filariasis elimination

States/Challenges	MDA								MMDP				Surveillance/Assessments			
	High Drug Refusal	Coverage Gaps (Urban)	MDA Supervision Gaps	Remote/ Inaccessible Areas	Never Treated Populations	Out- Migration	Gaps in Drug Distributor Training	Delays in IDA Rollout	Inconsistent Lymphoedema Grading	Hydrocele Surgery Backlog/Low Acceptance	Stigma and Poor Health Seeking	Delays in MMDP Kit Supply	In-Migration from Endemic Areas/Risk of Resurgence	Shortage of Testing Kits	Surveillance Gaps in Newly Developed Periurban Areas	Weak in Intersectoral Coordination
Northern region																
Uttar Pradesh	**✓**				**✓**	**✓**	**✓**	**✓**	**✓**	**✓**				**✓**		
Jharkhand	**✓**	**✓**	**✓**	**✓**	**✓**	**✓**				**✓**						
Bihar	**✓**	**✓**	**✓**	**✓**	**✓**	**✓**	**✓**		**✓**	**✓**						**✓**
Western, central, and eastern region																
Odisha		**✓**		**✓**						**✓**				**✓**		
Maharashtra	**✓**	**✓**														
Gujarat											**✓**		**✓**		**✓**	
Southern region																
Tamil Nadu													**✓**			
Kerala		**✓**											**✓**	**✓**		
Puducherry										**✓**	**✓**					
Karnataka	**✓**				**✓**	**✓**								**✓**		
Andhra Pradesh												**✓**	**✓**			

IDA = ivermectin, diethylcarbamazine, and albendazole; MDA = mass drug administration; MMDP = morbidity management and disability prevention.

In the eastern region represented by the state of Odisha, all districts continued to be endemic, contributing nearly 13% of the nation’s lymphoedema burden (82,136) and 17% of hydrocele cases (14,340). Ten districts had stopped MDA after attaining Mf rates below 1% since 2004. In the west, the state of Gujarat has 20 endemic districts, of which 14 have cleared TAS-3, indicating significant programmatic progress. The state of Maharashtra representing the central region has 18 endemic districts, with 8 having completed TAS-3.

The states/union territory representing the southern region were at a more advanced stage of elimination. Of the 38 districts in Tamil Nadu, 26 were previously endemic for LF, with the last MDA conducted in 2014 at 97.3% coverage. Currently, all endemic districts have completed all three serial TASs, and the state has submitted its elimination dossier for validation. Puducherry’s single endemic district had completed TAS-3 and is under post-MDA surveillance. Kerala has 11 endemic districts, of which 8 have completed TAS-3, whereas the remaining districts are preparing for TAS-1. Karnataka has nine endemic districts, with approximately 80% of cases concentrated in three districts, including Yadgir, Kalaburagi, and Bidar. Andhra Pradesh has 10 endemic districts with more than 40,000 lymphoedema cases. Eight districts have completed TAS-3. This regional variation provided important context for understanding the programmatic challenges discussed (Table 1) and underscores the need for region-specific strategic actions.

## KEY THEMATIC PRIORITIES AND POLICY DIRECTIONS FOR STRENGTHENING LF ELIMINATION IN INDIA

Seven thematic areas emerged from collective deliberations across the three workshops: 1) improving MDA coverage and quality; 2) strengthening MMDP; 3) addressing the never-treated (NT) population; 4) migration-focused strategies; 5) surveillance and diagnostics; 6) community engagement, information, education and communication (IEC), and behavior change; and 7) program management and financing. Each theme encompasses specific policy recommendations aimed at operationalizing India’s LF elimination commitment.

### Improving MDA coverage and quality.

The consultations identified improvements in MDA coverage and quality of implementation as key steps for accelerating LF elimination in India. MDA is implemented annually or biannually through a community-based system in India, in which frontline health workers and volunteers administer drugs, predominantly through a home-visit model. Accredited Social Health Activists (ASHAs) and Anganwadi workers (community-based childcare workers under the Integrated Child Development Services program) visit households to administer drugs.

Stakeholders from multiple states reported coverage issues. In Jharkhand, seasonal migration, drug refusals because of fear of side effects, and inadequate MDA supervision in rural areas undermined coverage. The state also reported significant coverage issues in urban areas, especially in housing societies and workplaces, where drug administrators have limited access. In Bihar, despite a WHO-monitored coverage of 67% in the 2025 round, 44 of 199 high-priority blocks recorded coverage below 45%. Karnataka and Maharashtra both reported high drug-refusal rates, with refusals in Karnataka mainly concentrated among elderly populations. Maharashtra has monitoring gaps in specific districts, including Nanded, Gadchiroli, and Chandrapur. In Odisha, coverage gaps were concentrated in periurban areas. Madhya Pradesh specifically needs better community awareness, stronger MDA supervision, and remapping of endemic blocks before MDA. Accurate coverage reporting is a further concern given that there is significant variation between program-reported figures and independent survey reports. This is particularly relevant as reported coverage remains the basis for program decisions of whether to proceed with epidemiological monitoring survey (EMS) or not.

Recommendations included stronger supervisory mechanisms during MDA rounds; the use of digital or real-time monitoring tools; and developing targeted microplans for areas with high-refusal rates, remote or hard-to-reach populations, urban slums, migrant clusters, and industrial zones. Discrepancies between the program coverage and the independent survey results indicate the need for stronger monitoring systems and improvements in actual drug uptake at the community level. The results of coverage evaluation surveys, which are sample surveys to validate the reported coverage, shall be used to make improvements in coverage. Strengthening the training for drug administrators and the rollout of the IDA regimen in newly identified hot spots were highlighted as essential components.

### Strengthening MMDP.

Gaps in MMDP were reported in all three regions. In northern states, backlogs in hydrocele surgery were substantial. Jharkhand recorded 40,000 hydrocele cases with high surgical backlogs, whereas Bihar reported more than 150,000 lymphedema cases and faced challenges in efficiently grading large numbers of patients. Uttar Pradesh reported low hydrocelectomy coverage relative to its burden. Odisha contributes approximately 17% of the national hydrocele burden, reporting challenges in patient management and service delivery. Maharashtra emphasized funding delays that disrupted MMDP services. In the southern region, Puducherry highlighted low patient uptake of both hydrocele surgery and MMDP services along with challenges in sourcing customized footwear locally. Nongovernmental organization partners noted program shortcomings, including low prioritization of MMDP at the primary health center level, delays in MMDP kit distribution, difficulties in procuring customized footwear, and difficulties in the implementation of disability certification for advanced lymphedema patients. The WHO representatives identified shortages of trained surgeons. Although national guidelines recommend annual enumeration and updating of patient lists, underidentification of patients because of stigma associated with the disease, shortages of field-level staff, and inconsistent enumeration by frontline workers were reported. In addition, limited training of medical officers in lymphedema grading contributed to delays in the appropriate classification of patients, which in turn, affected the timely implementation of disability certification in several states.

Interventions, such as standardized lymphoedema-grading protocols and dedicated training for medical officers, are particularly urgent in high-burden states, such as Uttar Pradesh, Bihar, and Jharkhand. Surgical backlogs in hydrocelectomy need to be reduced through dedicated surgical camps and systematic mapping of functional surgeons. A public–private partnership is essential to reduce the backlog of hydrocele surgeries. An uninterrupted supply of MMDP kits was recognized as an important requirement to improve MMDP care. Efforts must be made to motivate patients for surgery and improve their access to disability certification and existing social protection schemes. At the facility level, MMDP must be prioritized at the primary health centers through training and clearer accountability.

### Addressing NT populations.

Addressing NT populations was identified as a priority area. Karnataka specifically flagged the risk posed by NT and out-migrant populations as a major challenge for elimination given their potential to sustain transmission and remain outside program reach. The field experience of PCI in MDA rollout in Jharkhand showed the scale of the problem. They reported that nonparticipation in MDA rounds was as high as 40% in some villages owing to missed visits by administrators, fear of side effects, and mistrust in the community. Addressing NT populations is, therefore, both an epidemiological need and a matter of equity. Ensuring their inclusion is essential so that the benefits of the program reach everyone regardless of location or social status. The recommended strategies include targeted profiling and tracking of NT populations at village levels, intense social and behavioral change communication activities before and during MDA rounds, and training of drug administrators to tackle refusals at the community level. The recent NCVBDC guidelines also emphasize social and behavior change and advocacy as core strategies for building community readiness ahead of MDA.^2^

### Migration-focused strategies.

Population mobility was noted as a significant challenge for the LF elimination program in India. In northern states and Karnataka, the seasonal out-migration was listed as a reason for coverage gaps during MDA, whereas migration from endemic areas was noted as a major risk factor for reintroduction of infections in districts under post-MDA surveillance in the southern region. In 2024, screening of nearly 90,000 migrants in Tamil Nadu detected 246 Mf-positive cases, highlighting the risk of reintroduction of transmission. In Gujarat, it was highlighted that rapid urban expansion and mushrooming of informal settlements draws in large numbers of laborers from endemic states, such as Bihar, Uttar Pradesh, and Odisha, and the risk posed by this group is unknown in migrant pockets. Reluctance among migrants to undergo blood smear testing was a specific challenge for post-MDA surveillance in Puducherry. The consultations recommended migrant-specific strategies, such as tracking, testing, and treating migrant workers and implementing localized MDA camps in collaboration with the labor department and industries.

### Surveillance and diagnostics.

Robust surveillance systems are essential for early detection of residual or re-emerging transmission. Logistical hurdles, specifically delays in procurement and supply of rapid diagnostic kits, were identified as barriers to timely TAS and impact surveys. In Uttar Pradesh, shortages of diagnostic kits were reported as a challenge delaying pre-TAS activities. Kerala highlighted an urgent need for filariasis test strips (FTS) kits to initiate TAS activities and reported challenges in the distribution of *Brugia malayi*-specific kts in the Alappuzha district. Odisha similarly noted FTS kit shortages. Karnataka stressed the need for the timely distribution of diagnostic tests for surveillance activities. The representatives from the WHO also highlighted the shortages of *Brugia*-specific diagnostics and that *Brugia* Test Plus rapid diagnostic test kits (Drugs & Diagnostics for Tropical Diseases, San Diego, California, USA) are recommended by the WHO. However, the WHO has provided guidance on alternative strategies, including the use of Mf surveys, in situations of diagnostic kit shortages.^13^ Strengthening the availability of diagnostics through timely procurement is essential, particularly ensuring access to RDT kits in *Brugia*-endemic areas. Integration of vector surveillance using molecular xenomonitoring in high-risk and post-MDA districts and scaling up of vector control activities will be useful to stay vigilant of any resurgence in low-prevalence settings.^11^

### Community engagement, IEC, and behavior change.

The discussions noted that building public trust to increase participation in MDA and MMDP requires strong community engagement. Culturally sensitive IEC and behavior change campaigns are needed, particularly in high-refusal areas, in urban slums, and among elderly populations.^2^ In addition, special efforts are needed to address the social stigma associated with LF to promote timely care seeking and treatment adherence. Active involvement of NGOs, self-help groups (SHGs), and local influencers was identified as a measure to improve the acceptability of interventions. Many states demonstrated effective approaches in this regard. In Jharkhand, SHGs were engaged in providing drug consumption slips during MDA. The state also collaborated with NGOs, such as Piramal Foundation and Global Health Strategies, for community mobilization. Bihar used celebrity advocacy to build community awareness and conducted targeted campaigns in prisons, military areas, hostels, brick kilns, and nomadic settlements. Odisha engaged SHGs, the National Service Scheme, and youth groups during MDA. Tamil Nadu deployed 30,400 filaria-preventive assistants from NGOs, SHGs, Anganwadi workers, and social welfare and education departments for its MDA campaigns.

### Program management and financing.

The discussions highlighted the need for effective program management; timely location and disbursement of funds for activities, such as NBSs and TAS; and stronger intersectoral coordination across relevant departments. In India, the LF elimination program operates through a three-tier administrative structure. At the national level, the NCVBDC formulates the budget, mobilizes central government funding, issues guidelines on fund utilization, and distributes funds to states and union territories.^2^ MDA drugs are centrally procured by the NCVBDC and supplied to the states at no cost. At the state level, State Program Officers allocate funds to districts in accordance with central guidelines and consolidate expenditure reporting. At the district level, District Vector Borne Disease Control Officers further allocate funds to primary health centers and ensure utilization as per program guidelines. Participants highlighted some operational challenges, including fund shortages for NBSs and TAS activities, especially in Bihar, Uttar Pradesh, and Kerala. Inadequate drug administrator training and poor intersectoral coordination were noted in Bihar and Uttar Pradesh. Gaps in MDA supervision and community awareness in Madhya Pradesh were noted by the WHO country office. To strengthen program implementation, regular annual reviews at national and regional levels are recommended to monitor progress, identify gaps, and enable timely actions.

## REGION-SPECIFIC CHALLENGES

The challenges were discussed following each stakeholder’s presentation, and the outcomes of these deliberations were systematically summarized in Table 1. States in the north, such as Uttar Pradesh, Bihar, and Jharkhand, struggled with high LF endemicity; operationally challenging geography; an overburdened health system (the need to handle a huge population); and large seasonal out-migration that hinders efforts to achieve MDA coverage thresholds, mapping of patients, and surveillance, consistent with findings reported in previous studies.^14^^–^^17^ In central, western, and eastern regions, Maharashtra, Gujarat, and Odisha faced challenges related to urbanization and large inflows of unorganized migrants from endemic states under MDA as documented in the earlier literature.^18^^–^^21^ Southern states, including Tamil Nadu, Kerala, Karnataka, Andhra Pradesh, and the Union Territory of Puducherry, which are now largely in the post-MDA phase, faced the risk of resurgence because of increased population mobility from endemic areas. Previous studies in India have found high endemicity among the migratory population, highlighting their potential to cause resurgence.^22^^,^^23^ Across regions, participants highlighted gaps between program-reported and independently evaluated MDA coverage estimates, operational fatigue among frontline health workers, and low drug uptake in urban and periurban settings. The challenge of reaching out to people who have never taken MDA drugs was highlighted in line with the recent literature and WHO guidance.^24^^–^^26^ Barriers to MMDP, such as patient identification, inconsistent grading of lymphoedema, and access to hydrocelectomy and disability certifications, were also highlighted across regions, reflecting systematic constraints documented in the literature.^27^^–^^29^

## REMEDIAL MEASURES IDENTIFIED

A consensus was reached that social mobilization and trust-building activities would improve MDA acceptance in the community (Table 2). Active engagement with communities and leaders, capacity building of drug administrators, and the use of digital supervision tools during MDA were recommended. Real-time monitoring tools (e.g., the Sukritya app model in Bihar) were highlighted as effective tools for strengthening program accountability and enabling timely corrective actions when coverage gaps, missed areas, or missed populations are identified. Systematic profiling of NT populations was recommended as a component of block-level MDA microplanning, with NT tracking proposed as a reportable indicator within NCVBDC monitoring systems. Interpersonal communication, including peer demonstration strategies, public tablet consumption by ASHAs, and use of the locally familiar language, was recommended to improve acceptance. The involvement of NGOs, such as PCI in Jharkhand, to identify and reach NT populations through grassroots social mobilization is a replicable model for adoption in other high-burden states.

**Table 2:** Policy recommendations for accelerating lymphatic filariasis elimination

S. No.	Policy Area	Recommendations	Advantages/Benefits
1	MDA coverage and quality	● Strengthen supervisory visits during MDA ● Deploy digital supervision and real-time monitoring tools ● Targeted refusal-conversion strategies ● Targeted microplanning for urban slums, migrant clusters, hostels, and industrial zones ● Improve accuracy of coverage reporting ● Localized MDA for migrants in large clusters ● Targeted drives in low-coverage pockets ● Better reach to underserved/remote areas and mobile populations ● Enhance drug administrator training ● Accelerate IDA rollout in pending districts	● Real-time correction of operational gaps during MDA implementation ● Improves treatment coverage, and enhances compliance ● Faster reduction in infection levels
2	MMDP	● Better patient identification and care outcomes ● Standardized grading protocols for lymphoedema ● Training of medical officers on grading and MMDP ● Private partnership to reduce hydrocele backlog ● Strengthen counseling to increase surgery acceptance ● Improve supply chains of MMDP kits ● Lymphoedema patient follow-up and tracking system ● Strengthen disability certification and social protection schemes for advanced patients	● Better care for patients and prevents disease progression/disability ● Reduces hydrocele backlogs, and increases acceptance and uptake of surgical services ● Improves patient’s psychosocial well-being
3	Addressing NT populations	● Institutionalize NT profiling and tracking ● Introduce SBCC for chronic refusers ● Build capacity for refusal conversion	● Helps reach higher coverage, and eliminates hidden reservoirs sustaining transmission
4	Migration-focused strategies	● Track and treat migrant workers going out for work ● Surveillance of migrants in post-MDA districts ● Implement localized MDA for migrants with industrial partners	● Prevents reintroduction of infection in post-MDA districts, and maintains LF-free status
5	Surveillance and diagnostics	● Timely procurement and distribution of FTS/QFAT kits ● Intensified surveillance in high-risk migrant settlements ● Structured surveillance for new urban/periurban areas ● Strengthen xenomonitoring in post-MDA areas ● Scale up vector control in high-risk areas	● Facilitates earlier detection of emerging transmission hot spots for timely response ● Improves program evaluation ● Enhances detection of infection in vectors, and strengthens early response
6	Community engagement, IEC, and behavior change	● Intensive campaigns in high-refusal areas, among the elderly, in slums, etc. ● Scale up culturally sensitive IEC/BCC using local influencers ● Involve trusted NGOs and SHGs ● Sensitization to reduce stigma among patients	● Builds community trust, and increases participation in MDA and MMDP services ● Reduces stigma, and promotes timely care seeking
7	Program management and financing	● Dedicated budget for night blood surveys and TAS activities ● Improve intersectoral coordination	● Improves efficiency of program operations through better coordination and planning ● Ensures drug quality and safety

BCC = behavioural change communication; FTS = filariasis test strips; IDA = ivermectin, diethylcarbamazine, and albendazole; IEC = information, education & communication; LF = lymphatic filariasis; MDA = mass drug administration; MMDP = morbidity management and disability prevention; NGO = nongovernmental organization; NT = never treated; QFAT = Q Filariasis Antigen Test; S. = XXX; SBCC = social and behaviour change communication; SHG = self-help group; TAS = transmission assessment survey.

For morbidity management, the establishment of unique patient identifications and registries for lymphedema and hydrocele cases was recommended to ensure continuity of care alongside uniform patient-grading protocols. Direct linkage with disability pensions, transport subsidies, and livelihood schemes was emphasized as essential to integrating social protection within the elimination program. Addressing the challenge of the migrant population requires formal coordination between health, labor, municipal, and housing authorities, with LF control integrated into urban health mission and water, sanitation, and hygiene (WASH) initiatives. Partnership with industries, construction companies, and labor contractors was recommended to facilitate mandatory screening and ensure MDA coverage through targeted delivery mechanisms or a booth-based approach. Finally, strengthening vector surveillance efforts, especially in post-MDA districts/blocks, and regular supplies of diagnostics for TAS activities were suggested as essential requirements for the program.

## BEST PRACTICES

**Figure 2.**
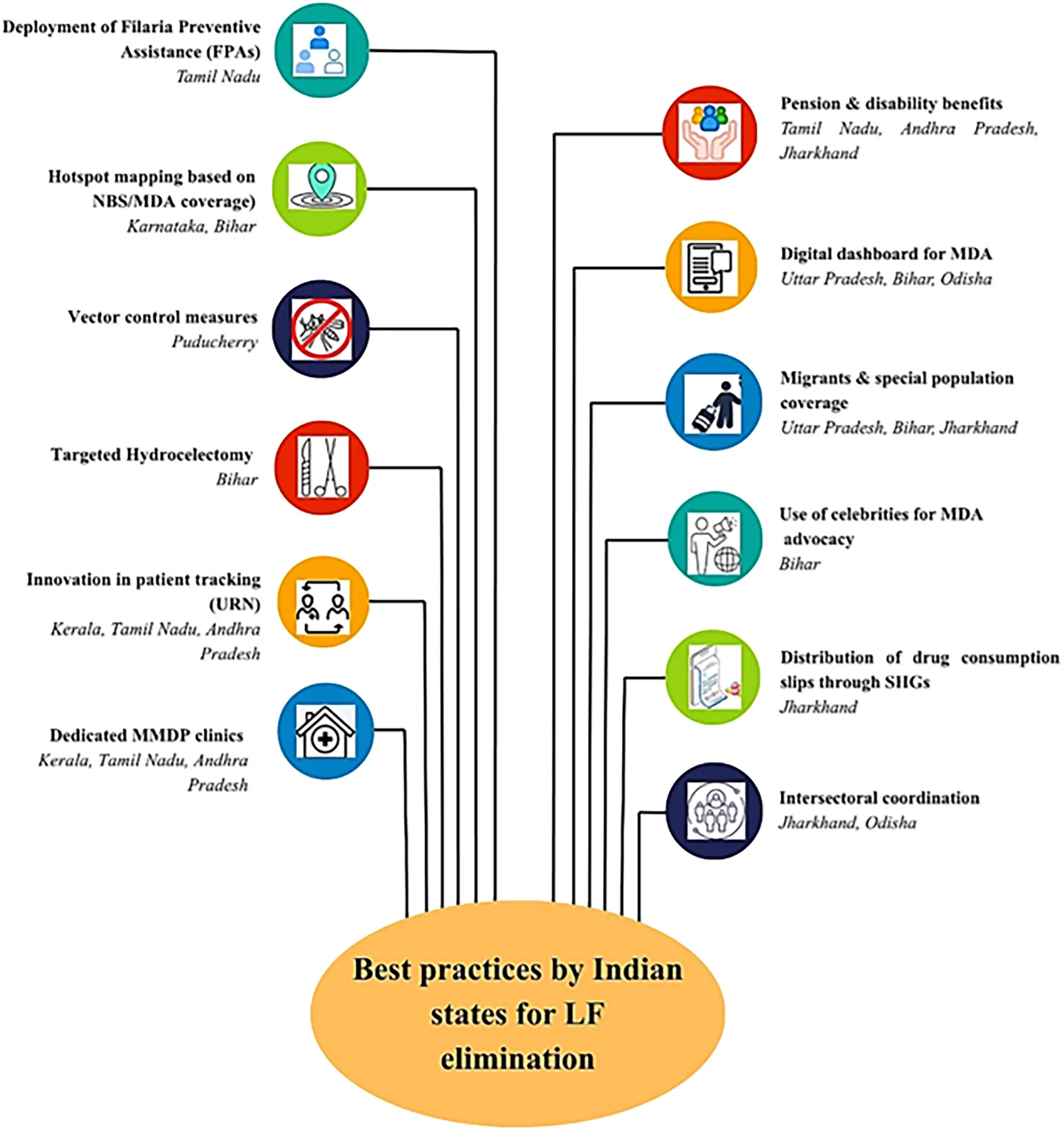
Best practices followed by endemic states toward lymphatic filariasis (LF) elimination in India. FPA = filaria preventive assistance; MDA = mass drug administration; MMDP = morbidity management and disability prevention; NBS = night blood survey; SHG = self-help group; URN = unified registration number.

Across regions, states have adopted a range of context-specific best practices to accelerate LF elimination (Figure 2). Jharkhand demonstrated strong multisectoral engagement through coordination across 24 departments; partnerships with NGOs, such as PCI, the Piramal Foundation, and Global Health Strategies; and the use of SHGs for distributing drug consumption slips. The state also undertook targeted MDA drives for vulnerable tribal populations. Bihar used a structured 3-day booth approach followed by 14 days of house-to-house visits. Celebrity advocacy was used to improve community acceptance, and daily MDA coverage was tracked in real time through the Sukritya mobile app. Odisha engaged SHGs in MDA delivery and instituted an automated real-time coverage reporting dashboard that enabled daily program monitoring. Social mobilization was strengthened through National Service Scheme volunteers, schools, and youth groups. The state also conducted refusal-conversion workshops for drug administrators to address community hesitancy. Maharashtra developed IEC materials in regional languages and maintained surveillance activities through night clinics and survey laboratories. In Gujarat, the state established strong entomological surveillance in urban areas. Tamil Nadu’s program was among the most comprehensively implemented in the country. The state deployed more than 30,400 filaria-preventive assistants drawn from diverse community platforms, a unique registration system for lymphoedema cases, monthly financial assistance for advanced-stage patients, and specialized physiomedical centers offering patient training and therapy. Kerala operationalized 83 MMDP clinics across the state, providing structured morbidity care to lymphoedema patients. Andhra Pradesh provides a monthly pension of rupees 6,000 to advanced-stage lymphoedema patients.

## CONCLUSION

The regional consultative workshops highlighted that although India has made significant progress, the situation differs by regions, demanding tailored strategies. States in the northern region continue to experience high infection levels, seasonal migration for employment, gaps in MDA coverage, and a significant number of NT populations. In the central and western states, rapid urbanization and the movement of migrant populations into areas close to elimination pose emerging risks. Eastern states continue to face operational challenges, particularly in reaching remote and difficult terrains. In the southern states, where most areas are in the post-MDA phase, there is an ongoing risk of reintroduction of transmission because of in-migration from endemic regions. These findings highlight the need for region-specific approaches, with greater focus on surveillance and stronger coordination across sectors to sustain progress and move toward elimination.
